# Supplementary material for: Sesamin Activates Nrf2/Cnc-Dependent Transcription in the Absence of Oxidative Stress in Drosophila Adult Brains
Source: Antioxidants (Basel). 2021 Jun 7;10(6):924. doi: 10.3390/antiox10060924 (PMC8227698; doi:10.3390/antiox10060924)
Supplement: Supplementary file 1 [file antioxidants-10-00924-s001.zip › antioxidants-1215140-SI.pdf]

## Supplementary Information

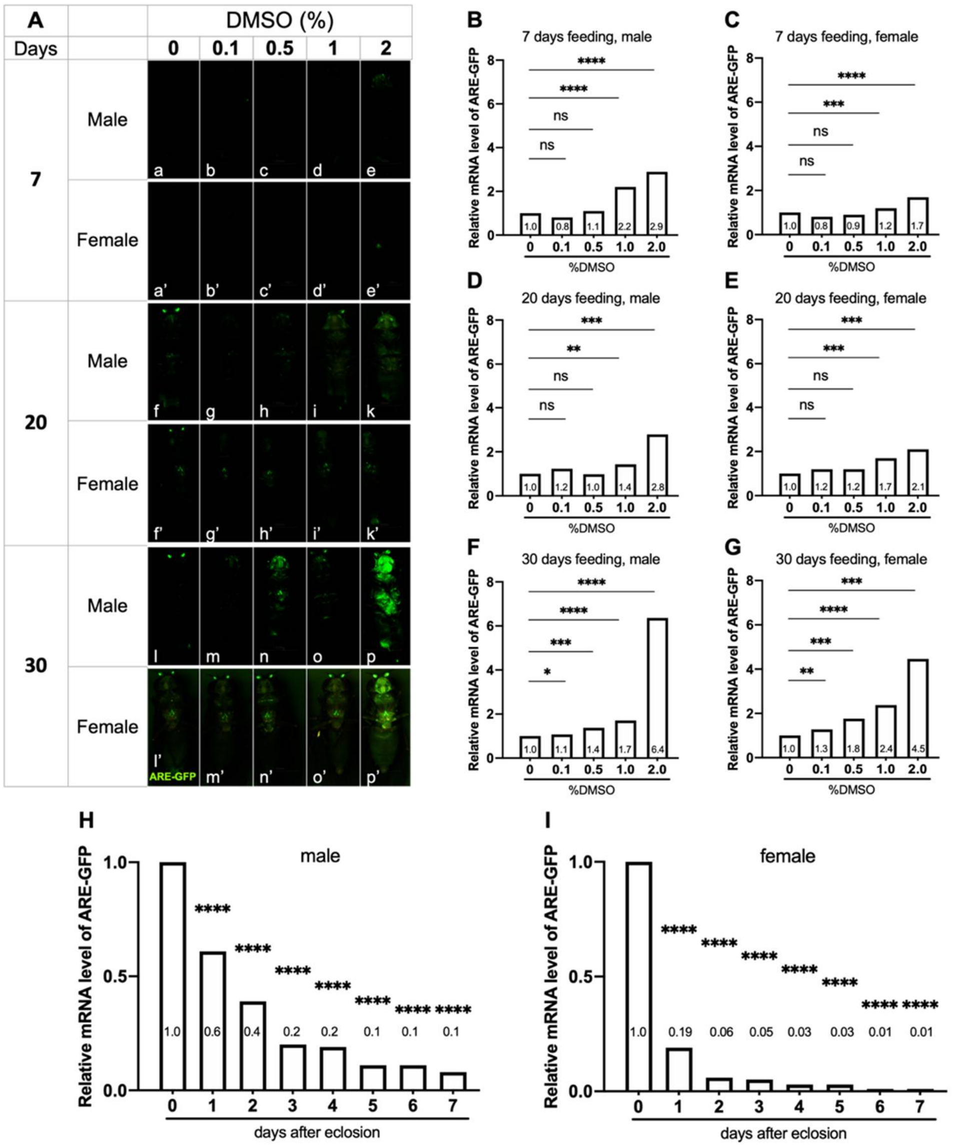


**Figure S1.** ARE-GFP reporters depend on DMSO concentration. (**A**) ARE-GFP reporter flies fed on the five different DMSO concentrations: 0% (as a control) or 0.1%, 0.5%, 1% and 2% for 7 days, 20 days or 30 days. (**B-G**) The mRNA level was quantitated by qRT-PCR using total RNA extracted from 10-15 whole bodies under DMSO concentrations: 0% (as a control) or 0.1%, 0.5%, 1% and 2%: at 7 days of male (B), of female (**C**); at 20 days of male (**D**), of female (**E**); at 30 days of male (**F**), of female (**G**) respectively. (**H-I**) The GFP mRNA level was quantitated by qRT-PCR using total RNA extracted from 10-15 whole bodies in the standard diet from 0-7 days of male (**H**), of female (**I**). Average mRNA levels were calculated from results of more than three independent qRT-PCR experiments. Statistical Analysis were performed by Student's *t*-test (**B-G**), one-way ANOVA (**H-I**), ns not significant, *$p < 0.05$, **$p < 0.01$, ***$p < 0.001$, ****$p < 0.0001$.

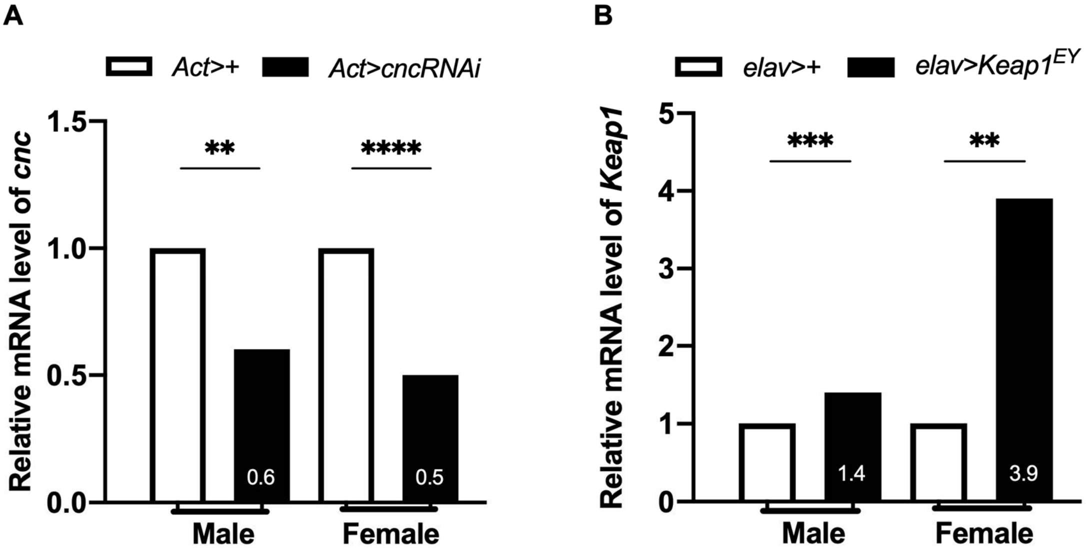


**Figure S2.** qRT-PCR to confirm depletion of *cnc* and overexpression of *Keap1*. (**A**) Ectopic expression of dsRNA against *cnc* mRNA using *Act-Gal4* driver (*Act>cncRNAi*) can deplete endogenous *cnc* mRNA in adult whole bodies efficiently. (**B**) Overexpression of *Keap1* in adult brains using a pan-neuronal Gal4 driver, *elav-Gal4* in the adult brain (*elav>Keap1*$^{EY}$). The mRNA level was quantitated by qRT-PCR using total RNA extracted from 10-15 whole bodies (**A**) or 40-50 heads (**B**). Average mRNA levels were calculated from results of more than three independent qRT-PCR experiments. Statistical Analysis were performed by Student's *t*-test, ***p* < 0.01, ****p* < 0.001, *****p* < 0.0001.

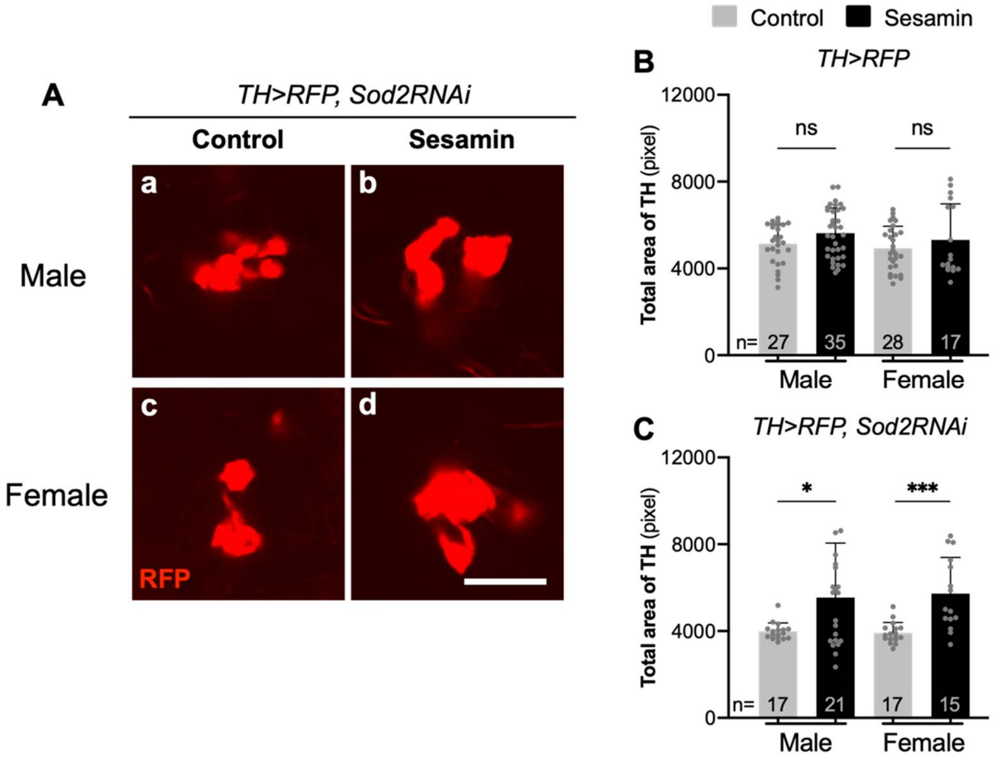


**Figure S3.** Suppression effects of sesamin on the oxidative stress-induced reduction of areas occupied by dopaminergic neurons in adult brains harboring dopaminergic neuron-specific depletion of *Sod2* in adult brain. (**A**) In dopaminergic neurons labelled in red by RFP expression, and the neuron-specific depletion of

*Sod2* mRNA was carried out *(TH>RFP, Sod2RNAi)*. Only a basal level expression of ARE-GFP were observed in clusters of dopaminergic neurons in male brain (**Aa**) and female brain (**Ac**) from flies fed on the diet, while feeding of the diet with sesamin at 2 mg/mL enhanced the GFP fluorescence in both male (**Ab**) and female brains (**Ad**). (**B, C**) Total area occupied by the dopaminergic neurons in brain *(TH>RFP)* (**B**), or in brains harboring dopaminergic neuron-specific ROS accumulation *(TH>RFP, Sod2RNAi)* (**C**) from adults ($n \geq$ 15 each). Brains were prepared from the male or female flies fed on food either with 0.5% DMSO only (control), or with sesamin at 2 mg/mL in 0.5% DMSO (sesamin) for 7 days. Scale bar represents 20 μm. Student's *t*-test, ns not significant, *$p < 0.05$, ***$p < 0.001$.
